# Linguistic Validation of a British-English Version of the SAMANTA Questionnaire and HMB-VAS Tool: A Step Toward Improved Diagnosis of Heavy Menstrual Bleeding

**DOI:** 10.1089/whr.2024.0061

**Published:** 2024-12-10

**Authors:** Josep Perelló-Capó, Joan Rius-Tarruella, Joaquim Calaf Alsina

**Affiliations:** ^1^ Hospital de Sant Pau, Barcelona, Spain.; ^2^ Department of Paediatrics, Obstetrics and Gynaecology, Preventive Medicine and Public Health, Universitat Autònoma de Barcelona, Barcelona, Spain.; ^3^ Bayer Hispania, Barcelona, Spain.

**Keywords:** heavy menstrual bleeding, abnormal uterine bleeding, linguistic validation, questionnaire, SAMANTA questionnaire, HMB-VAS tool

## Abstract

**Background:**

Heavy menstrual bleeding (HMB) is a common disorder interfering with physical, emotional and social domains, and overall quality of life (QoL). The Heavy Menstrual Bleeding-Visual Analog Scale (HMB-VAS) tool, including the VAS for menstrual bleeding intensity (VAS-Int) and the VAS for its interference with daily activities (VAS-Imp), is useful for HMB screening. The SAMANTA questionnaire (SAMANTA-Q) was developed and psychometrically validated in Spanish to easily identify women with HMB. However, these instruments have not been validated in other languages. This study seeks to linguistically validate the SAMANTA-Q and the HMB-VAS tool in British English.

**Methods:**

Linguistic validation was conducted following the principles laid out by the International Society for Health Economics and Outcomes Research (ISPOR). This process included two forward translations by two independent native English-speakers; reconciled version; two backward translations by two independent native Spanish-speakers; review and reconciliation; 60-minute cognitive debriefing interviews with women with HMB balanced by educational levels; analysis and integration of changes in the reconciled version; proofreading, and creation of the final version.

**Results:**

No major issues were found in the linguistic validation process. Overall, cognitive debriefing participants with HMB considered that the SAMANTA-Q and the HMB-VAS tool were easy to read, comprehensive, quick to answer, and covered most of the issues related to HMB.

**Conclusions:**

Linguistically validated British-English versions of the SAMANTA-Q and HMB-VAS tool are now available for clinical practice and research. These validated tools will be useful to easily diagnose excessive menstrual blood loss impacting on QoL.

## Introduction

Heavy menstrual bleeding (HMB) significantly affects women’s quality of life (QoL), with a prevalence of 30% among reproductive-aged women, increasing with age.^1–5^ Typically defined as blood loss exceeding 80 mL per cycle, HMB leads to iron-deficiency anemia in 25% of women affected.^6–9^ Besides physical symptoms due to anemia-associated blood loss (fatigue, lethargy, exertional dyspnea), HMB is often the initial symptom of uterine conditions, vascular abnormalities, malignancies, or coagulation disorders, among others.^10–12^ In addition, HMB negatively affects various aspects of women’s lives, including work, social life, relationships, and daily activities; with severe repercussions on QoL.^13–18^ Apart from its clinical and personal burden, HMB entails substantial economic costs due to healthcare resource utilization and productivity loss.^2,19–21^ Addressing HMB should prioritize improving women’s QoL alongside managing blood loss.^5^

Currently, a range of objective measures for estimating menstrual blood loss are available: laboratory-based quantitative methods, (alkaline hematin), and semi-quantitative methods, such as menstrual diaries or the Pictorial Blood loss Assessment Chart.^22–24^ Nevertheless, the alkaline hematin technique is expensive, laborious, and diaries/pictorial methods require an observational period, which makes them burdensome. Furthermore, all current measures are time-consuming.^22,23^ Importantly, none of the tools consider the impact of HMB on QoL. Moreover, these tools perform better in controlled clinical settings and their lack of standardization hinders their widespread use. Patient perception of blood loss severity seldom correlates with reality, this perception differs depending on an individual’s sociocultural and ethnical characteristics.^25^ For daily clinical practice, easy-to-use validated questionnaires are needed, considering not only blood loss, but also its impact on QoL, that is, the patient’s perspective.^24^

Patient-reported outcome measures (PROMs) are designed to directly obtain information from patients regarding clinical outcomes, treatment efficacy, and patient QoL, thus facilitating more personalized management and follow-up.^26^ For HMB assessment, unidimensional questionnaires considering a single domain (menstrual symptoms, QoL, or QoL in HMB) and multidimensional questionnaires including combined detection of menstrual symptoms and QoL impairment due to HMB, are available.^24^ However, some are excessively long,^27–30^ culturally sensitive,^31^ or more focused on detecting associated hemostatic disorders than HMB’s impact on womens’ lives.^32^ Validated tools are essential to accurately detect HMB volume and its impact on QoL, bridging the gap between physicians’ and patients’ perspectives.^33^ Although most HMB studies use generic indicators for QoL,^14^ we have developed a 6-item instrument to easily identify women with HMB interfering in their QoL: the SAMANTA questionnaire (SAMANTA-Q).^34^

SAMANTA-Q is an interviewer-administered tool that has been psychometrically validated in Spanish, demonstrating a high accuracy, identifying women with HMB (true positives; sensitive) and women without HMB (true negatives; specific). SAMANTA-Q combines the assessment of blood loss with the impact of HMB on QoL.^34^ It has been validated as a combined Heavy Menstrual Bleeding-Visual Analog Scale (HMB-VAS) tool, which assesses the intensity of menstrual bleeding (VAS-Int) and its impact on the activities of daily living (VAS-Imp) to identify women with HMB. It demonstrated accurate performance during HMB screening, providing an easy-to-use alternative to other psychometric tools.^35^ Moreover, the HMB-VAS tool was psychometrically evaluated, demonstrating a strong correlation with the SAMANTA-Q scores (*p* < 0.001).^34^

These instruments, currently only validated in Spanish, could greatly benefit from linguistic validation in other languages. Expanding translations would enhance accessibility for women with HMB, improving their care and QoL. In addition, as English is the language of scientific research, translating these instruments into British English could offer numerous professional opportunities and facilitate global dissemination of knowledge. Hence, this study aimed to linguistically validate these instruments in British English, benefiting both women with HMB and the scientific community.

## Methods

### Design

**FIG. 1.**
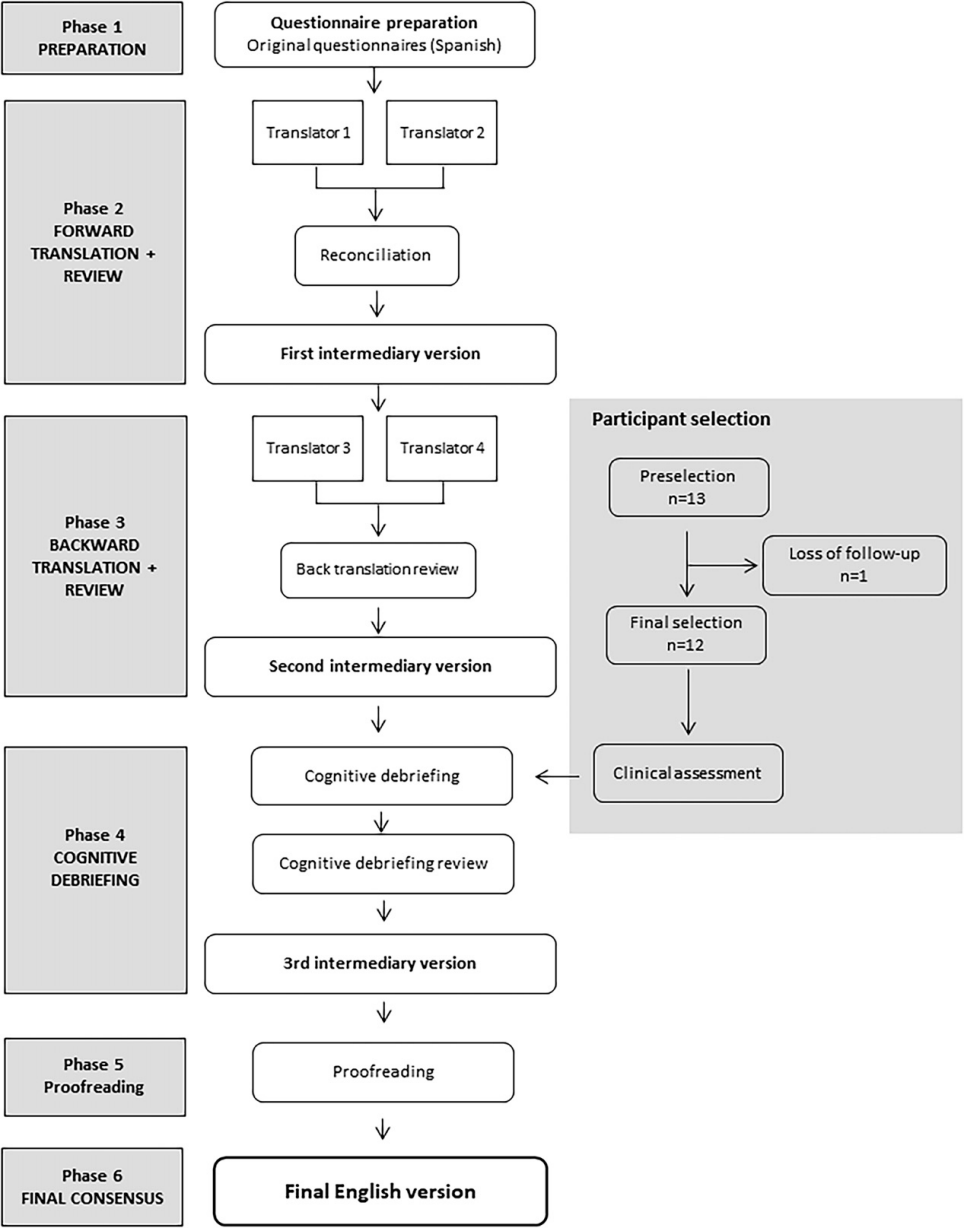
Flow diagram of the linguistic validation process into British English of the SAMANTA questionnaire and HMB-VAS tool, combining the intensity of menstrual bleeding scale (VAS-Int) and the interference of menstrual bleeding in daily activities scale (VAS-Imp). HMB-VAS, Heavy Menstrual Bleeding-Visual Analog Scale.

A qualitative, nonclinical, noninterventional, linguistic validation study was conducted adhering to the principles of the International Society for Health Economics and Outcomes Research (ISPOR) guidelines.^36^ The study comprised nine steps: 1) preparing study documents (screener, interview guides, data recording templates, participant consent and data protection documents); 2) forward-translation by two independent English-native translators, 3) reconciliation to create a merged single “forward translation,” 4) back-translation by two independent Spanish-native translators, 5) back-translation review, 6) cognitive debriefing (conducted by a native English interviewer to assess understandability, interpretation, and cultural relevance of the translation), 7) cognitive debriefing review (analysis and interpretation of the cognitive interview results to highlight and amend discrepancies), 8) proofreading (final review to highlight and correct any typographic, grammatical, or other errors), and 9) final version creation of the questionnaire and HMB-VAS tool in British English (Fig. 1).

### Measuring instruments

The Spanish original version of the SAMANTA-Q is composed of 6 items, all with binary response options (Yes/No). Affirmative answers to items 1 and 3 are assigned a score of 3 points, while affirmative answers to items 2, 4, 5, and 6 are each assigned a score of 1 point. Negative answers are scored 0. Thus, the final score of the questionnaire ranges between 0 and 10, with a total score ≥3 indicating that the woman may, or do, have HMB.^34^ SAMANTA-Q demonstrates both high sensitivity (86.7%) and specificity (89.5%), and good sensitivity to change after 1 year.^34,37^

The HMB-VAS tool includes two scales: VAS-Int and VAS-Imp. Both VAS scores are depicted on a 100-mm horizontal line representing a continuum between “Not bleeding at all” (0) and “The heaviest possible menstrual bleeding I have had” (100) for VAS-Int, and “Does not interfere in my daily life/activities at all” (0) and “Completely interferes in my daily life/activities” (100) for VAS-Imp.^35^ Total HMB-VAS score is obtained from this formula: 10.9 × VAS-Int score + 2.5 × VAS-Imp score, and has a cut-off point of 700. The HMB-VAS tool was psychometrically evaluated showing both high sensitivity (89.6%) and specificity (85.0%).^35^ The original Spanish versions of the SAMANTA-Q and HMB-VAS tool (VAS-Imp and VAS-Int) can be found in Supplementary Appendix S1.

### Procedure

For the linguistic validation process, the original Spanish SAMANTA-Q and the HMB-VAS tool, including the VAS-Int and the VAS-Imp, were translated into British English by two independent English-native translators (one female and one male to ensure representation of both genders). These translations were then compared with each other and with the original Spanish version. Discrepancies were resolved and merged into a single “forward translation” version, called the 1st intermediary version (Fig. 1). Subsequently, the 1st intermediary version was translated back into Spanish by two independent Spanish-native translators (also one female and one male). The back-translated versions were compared with each other and with the original Spanish version to identify discrepancies as well as to achieve a consistent approach to translation differences. Any discrepancies were addressed by the forward-translated version, resulting in the creation of the 2nd intermediary version (Fig. 1). This 2nd intermediary version was then used for the cognitive debriefing process.

Cognitive debriefing interviews were conducted using the online platform Zoom. During interviews, we applied a semi-structured interview guide and a “thinking aloud” method. The interview outline used can be found in Supplementary Appendix S2. In the “thinking aloud” step, each participant completed the questionnaire by reading all instructions, items and response options while verbalizing their decision-making process, providing insights into their comprehension of each element and the mental process behind their answers. The investigator took notes to record observations and to highlight areas for further inquiry. Before starting, participants were informed that they could leave the interview at any time, interrupt it at any time, or refuse to answer a question. Then, each participant was shown a questionnaire and asked to complete it. Following the completion of the “thinking aloud” process, a full cognitive debriefing interview was conducted, during which participants were asked detailed questions regarding each element of the questionnaire. This standardized approach to cognitive debriefing was carried out following the Willis method.^38^ Interviews were audio-recorded to be kept as a source document. All interviews were conducted in line with ISPOR guidelines.^36^ Importantly, the study did not interfere with any investigator’s decision regarding the most appropriate treatment/s for their participants, nor did it expose participants to any treatment.

### Participant selection

Screening for potential candidates was conducted, starting on September 6, 2023, and concluded on October 9, 2023. The recruitment period ended upon reaching the target number of participants. Inclusion criteria were: age of ≥18 years, HMB diagnosis in the previous 24 months, native British English speaker, with no special consideration regarding current or previous treatment, without malignant disease, able to participate with and understand the aim of the cognitive interview, capable of reading and writing, and willingness to sign the informed consent form. Exclusion criteria were: amenorrhea, menopause, pregnancy, women who had given birth within the previous 6 months, mental illness, or inability to make decisions or follow instructions. Participants were recruited using market research methods, through a fieldwork company in the United Kingdom (Sermo).

Potential candidates completed a clinical assessment interview with the Scientific Advisor (J.P.) to assess their eligibility. The Scientific Advisor collected data regarding their age and education level and confirmed the HMB diagnosis. Selected candidates were asked to attend an online cognitive debriefing interview with a native English interviewer to test the 2nd intermediary version of the questionnaire, to assess the understandability, interpretation, and cultural relevance of the translation and to explore alternative wording in case it was needed. One trained interviewer conducted cognitive debriefings and one consultant expert in linguistic validation processes managed the process of reconciliation of the different versions. The cognitive debriefing process took place between October 2, 2023, and October 11, 2023.

Before starting the interview, oral informed consent was obtained and recorded for all participants. Interviews were audio-recorded to be kept as a source document.

### Ethical considerations

Ethical approval for this linguistic validation study was obtained from the Ethics Committee for research with medicinal products (CEIm) from Hospital de la Santa Creu i Sant Pau (Barcelona), reference HSCSP 23/233.

## Results

### Forward translations

The two versions of the forward translation of SAMANTA-Q are presented in Supplementary Table S1, together with the single “forward translation” (1st intermediary version). Both forward translations demonstrated close coincidence, and most terms applied in the First version were chosen to preserve the original concept and convey the intended meaning accurately. Minor changes were applied for consistency reasons.

The two English versions of the forward translation of the VAS-Int are shown in Supplementary Table S2. Both forward translations showed a high degree of similarity, with only minor modifications considered. Likewise, the two English versions of the forward translation of the VAS-Imp are shown in Supplementary Table S3. Again, both forward translations were remarkably consistent and when the translations differed, the second translation was adopted to ensure coherence with the original scale.

### Back translations

Next, the reconciled forward translation (1st intermediary version) was translated back into Spanish by two independent Spanish-native translators. The two versions of the back translations were compared, and, in case of discrepancies, changes were incorporated into the forward translated version of the questionnaire, thus obtaining the “2nd intermediary version.” Most changes were applied to maintain the original concept and to ensure that the intended meaning was accurately reflected. Minor changes were also applied for consistency reasons.

Back translations for the 2nd intermediary version of the VAS-Int are shown in Supplementary Table S2. Most changes were applied to accurately reflect the original Spanish wording, and minor changes were also applied for consistency reasons. Back translations for the 2nd intermediary version of the VAS-Imp are shown in Supplementary Table S3. Changes were carried out to better align with the original Spanish wording and minor changes were also applied for consistency reasons.

### Cognitive debriefing

A total of 13 participants fulfilling inclusion/exclusion criteria were enrolled in the study; however, only 12 participants completed the interview and were finally included (Fig. 1). One participant was enrolled but failed to attend the scheduled evaluation appointment. Participants’ ages ranged between 24 and 45 years, with a mean of 34.83 (SD 7.26) years. A balanced educational level was observed (Table 1). All participants were diagnosed with HMB, as per inclusion criteria.

**Table 1.** Characteristics of the Participants Included in Cognitive Debriefing Interviews

Characteristics	*N* = 12
Age (years)	
mean (SD)	34.83 (7.26)
median (min; max)	35 (24; 45)
HMB diagnosis, *n* (%)	12 (100%)
Time from HBM diagnosis	
Within the last 12 months, *n* (%)	8 (66.67%)
Between 12–24 months ago, *n* (%)	4 (33.33%)
Educational level	
Master’s Degree or equivalent, *n* (%)	4 (33.33%)
Bachelor’s degree or equivalent, *n* (%)	3 (25%)
A-Level or equivalent, *n* (%)	3 (25%)
GCSE or equivalent, *n* (%)	2 (16.67%)

GCSE, general certificate of secondary education; HMB, heavy menstrual bleeding; SD, standard deviation.

Participants’ opinion/feedback on the understandability, interpretation, and cultural relevance of the SAMANTA-Q and the HMB-VAS tool were recorded by the interviewers and are showed in Tables 2–4.

**Table 2.** Participants’ Opinions Expressed during Cognitive Interviews. SAMANTA Questionnaire

Second intermediary version	Participants’ opinions	Third intermediary version
Do you bleed for more than 7 days every month?	Clear question, easy to answer rapidly for most. For some (especially more irregular periods) lack of precision about cycles question refers to *e.g.*,: last few periods, last 4–6 periods, usually, generally, sometimes, *etc.* Issue easy to address, with minor change. Well understood generally, but some wonder if it means “7 consecutive days” of one’s period, or if it also includes spotting or bleeding during ovulation. They wonder whether to include ovulation days plus their period days, and are unsure how to answer, *e.g.*, Yes — 5 + 2 days, or No — just 5 period days.	Generally, do you bleed for more than 7 days every month?
Do you have 3 or more days of increased heavy bleeding during your period?	Clear for most and considered easy to answer. Most words found relevant and easy to understand (*i.e.*, “3 or more days,” “heavy bleeding,” “during your period,” “period”). Good identification with HB on 3 days of their period, or 2–3 days (min.) Some difficulty understanding “increased heavy bleeding,” and differentiating it from “heavy bleeding,” probably because it’s a phrase, they’ve never heard before. When they don’t understand it, tend to ignore it/skip over it, but feel they can still answer the question by interpreting it as referring to “heavier bleeding,” on 3+ days period. Just “Heavy bleeding” or if comparative “heavier bleeding” is more usual vocabulary. “Increased heavy bleeding” intuitively seems to mean “more” than heavy bleeding, (*e.g.*, “more than the day before”). Spontaneous vocabulary played back—really heavy, very heavy, extremely, extra-/super-heavy, severe, “flooding,” “soaking” *etc.*—but these terms are not generally perceived as better alternatives to “increased.”	Do you have 3 or more days of increased heavy bleeding, during your period?
In general, do you find your periods particularly inconvenient due to their heaviness?	Straightforward question. Clear and easy to understand. Emphatic/relevant vocabulary: “particularly inconvenient,” “due to their heaviness”—appreciated, strong identification with interference/disruption caused by heavy periods. “Period” typical vocabulary. Understood as “that time of the month!”/when bleeding during menstrual cycle. They’re so used to using “period,” often can’t think of a synonym (except for cycle). “In general,” is a useful clarification which is welcomed.	In general, do you find your periods particularly inconvenient, due to their heaviness?
On any of the heavier bleeding days, do you bleed and stain your clothes during the nights, or would you stain them if you did not use double protection or change during the nights?	Long and unnatural feel may seem strange to some; a few recommend splitting it for clarity. However, most participants grasp the question upon the second, if not the first reading, and are comfortable answering it. Key vocabulary—relevant, clear: “bleed,” “stain,” “during the night,” “double protection.” Although “leak” is more commonly used in place of “bleed”/“stain” and frequently played back spontaneously. Identify well with 2nd part (from “or”). They’re using double protection, getting up to change more than once in the night, even so leaking (which implies staining) can occur and is a significant concern—“preys on your mind.” (Although, this is true for both day and night-time). Grammar error “nights” picked up actively by some, others latently (*e.g.*, miss off “s,” or say “those nights,” naturally), “the nights” should be “the night” (without an “s”). “During the nights” sounds odd. Often play back “those nights” or “the night” when reading question out loud/talking about it. Also, “during the nights” appears twice, repetitive. “Clothes” generally indicates daywear, not nightwear. Patients’ playback: pyjamas, nightwear *etc.* Note: It’s more usual to talk about nightwear for clothing worn in bed. Bed-clothes (sheets…) are felt to be missing from question—staining sheets more problematic than pyjamas. Suggestions “stain your nightwear/bedding.” Note: a bit contradictory in talking about heavier bleeding days… during the nights… This may account for some of the dissonance/"strangeness.” Note: Negative conditional clause “would… if you did not” makes question longer, more complex/a bit harder to understand in one go. Note: Preference for affirmative non-conditional clauses-Plain English Campaign (*e.g.*, “Are you concerned about staining your clothes and bedding during the night?”). Some suggest including “daytime” too. Using double-protection, frequent changing, fear of leaking, “accidents,” staining clothes not.	On any of the heavier bleeding days, do you bleed and stain your nightwear during the nights, or would you stain it, if you did not use double protection or change during the nights?
During heavier bleeding days, do you worry about staining the seat of your chair, sofa, *etc.*?	Simple direct. Easy to understand and answer. Language appropriate (“heavier days,” “worry,” “staining”). Note: “leaking” played back/ more usual term. Good identification, especially with leaking outside home (In-home lesser concern). Most believe question is asking about staining seats outside home (work, car, public transport, restaurants). Although, non-working women sometimes think it refers to home only: “because it says “sofa.” A suggested shorter version would be just saying ‘your seat *etc.*?’ (*i.e.*, remove “chair, sofa,” while others prefer to maintain these examples).	During heavier bleeding days, do you worry about staining the seat of your chair, sofa, *etc.*?
In general, on heavier bleeding days, do you avoid (as much as possible) some activities, travel or leisure plans because you need to change your tampon or pad frequently?	Feels a long question, several clauses. Some have to read it twice. Positive vocabulary included: avoid, tampon or pad (preferred to more formal “sanitary protection”). “Avoid”—appropriate word is “stop doing.” Use of avoid debated because it can feel too emphatic. They say they plan around their periods due to their unavoidable commitments—work/ meetings/journeys/ child-care/school run/shopping *etc.* Avoid can also imply turning your back on a normal life—undesirable, even for those who “try not to leave house for 2 days.” Alternatives explored: limit, restrict—change meaning slightly, and not felt to have better fit. Suggested addition “try to” overcomes problem and is often preferred (prompted). Sounds more natural, with better fit for working around/ planning for HB, as much as they can. “As much as possible” felt wordy caveat. Also, “extreme meaning” for those with less heavy HB but appropriate for “extreme HB patients.” “When possible”/where possible/“if possible”/"when you can/if you can” are shorter, with similar but slightly softer meaning. Or if “try to” is used, could consider omitting caveat without changing meaning significantly. “Some activities” was understood and accepted by most, despite more formal register. However, some think question asking about very active sports (whitewater rafting, trampolining) rather than mundane everyday activities like school-run, going shopping, catching bus/train, going to work, having lunch out, going swimming *etc.* “Daily life”/everyday life/day to day life *etc.* used spontaneously and often preferred by majority. A compromise solution would be to include “daily” before activities to limit differences with original text.	In general, on heavier bleeding days, do you avoid (as much as possible if possible) some certain activities, travel, or leisure plans, because you need to change your tampon or pad frequently?

**Table 3.** Participants’ Opinions Expressed During Cognitive Interviews of the Visual Analog Scale Scores for the Intensity of Menstrual Bleeding

Second intermediary version	Participants’ opinions	Third intermediary version
INTENSITY OF MENSTRUAL BLEEDING-VISUAL ANALOGUE SCALE -	Good, well-understood means severity/volume of flow. (It could also apply to pain but clarified below—“heaviness”/volume). Subtitle: “Visual analogue scale”—less understood, *e.g.*, misread as “virtual” or reject “visualizing” their heavy bleeding (minority). Often overlooked. “Scale,” a more usual term, played back by some. Some participants suggest removing “visual analogue scale” in the title since it is considered redundant.	INTENSITY OF MENSTRUAL BLEEDING VISUAL ANALOGUE SCALE
Please assess the intensity of your menstrual bleeding.	Terminology: Good appropriate, easy to understand, *i.e.*,: Assess, Intensity, Menstrual Bleeding. However, incomplete (for some). Query which cycle(s) to assess: this one, last one, last few (3, 4, 6 cycles) average, usual MB, *etc.* A timeframe or time/frequency adverb would overcome this. (Especially if more irregular periods/“recent” HB > don’t know how to include irregular/ lighter periods in assessment “have to” make assumptions. Note: used timeframes in health questionnaires *e.g.,* “Thinking over your last 4 periods” *etc.*, but this would differ from original.	Please assess the intensity of your menstrual bleeding, generally
Mark a vertical stroke on the following line to indicate the intensity of your menstrual bleeding.	Instruction understood, but “mark a vertical stroke” surprising term. “Mark a vertical stroke” unfamiliar lexicon, “put a cross” more usual and preferred option. “Mark with a cross” also acceptable. “Draw a line” is the preferred alternative (if there’s a need to maintain “line,” for linguistic consistency). “Vertical” a difficult word “for others who don’t have good English,” although they all understand it. “Vertical” can also feel superfluous—implicit (min.) The second part makes perfect sense, *i.e.*, “on the following line to indicate the intensity of your menstrual bleeding”	Option 1: Draw a vertical line on the following line to indicate the intensity of your menstrual bleeding. Option 2: Draw a vertical line on the following line to indicate the intensity of your menstrual bleeding.
Not bleeding at all	Easy to understand. Find it logical that “0” should be “not bleeding at all”—accepted for a 0–100 scale. “No bleeding at all” played back. “At all”—“unnecessary’ on “zero” scale, remove it and shorten question is suggested. Some participants pointed out that “Not bleeding at all” = “no blood,” meaning “not menstruating,” so not actually talking about their periods/ days of bleeding. Could refer to “menopause” *etc.*	“0” Not bleeding at all
The heaviest possible bleeding I have seen	Initially, this scale doesn’t sound/feel quite right. It can be understood but slightly problematic. “Heaviest possible bleeding” is imprecise. Unclear which cycle referring to:—average, usual, current/now, last cycle, last few cycles, *etc.* More precision wanted (irregular periods *etc.*) *i.e.*, timeframe or frequency adverb, *e.g.*, generally. Suggests extreme bleed outs: terrorism, road accidents, miscarriage, hemorrhage. Adding the word “menstrual” overcomes this. “Heaviest possible bleeding”—possible is before the noun, does not sound/feel quite right for some. “Possible” can be interpreted as maximum possible or “may be possible”/"could be” or “imaginary,” *i.e.*, not always understood as superlative “the most.” Note: positioning “possible” after noun may resolve this. It may change feel and meaning slightly, *i.e.*, “heaviest bleeding possible”— emphatic superlative [*i.e.*, “which possibly could exist”]. Also, no verb required, (*i.e.*, can remove “I have seen”). “I have seen” not the “right” expression in English and can feel illogical and subjective. Preferred alternatives played back: “had”/–“experienced”—more usual and natural way to express “I have seen” in this context. HMB is not observed/visualized (in others or ‘selves) but felt and experienced personally. Often, unsure what they should be comparing visually—seeing their own period vs. looking at other women’s periods? Or more universal “heaviest possible in the world.” Some participants suggested an objective comparison would be number pads/ tampons used on heavy days—*i.e.*, not experienced nor seen, but measured/ counted. It is suggested to use reduced scale 0–10, similar to pain scale, felt easier, more familiar.	Option 1: “100” The heaviest menstrual possible bleeding possible Option 2: “100” The heaviest possible menstrual bleeding I have had seen

**Table 4.** Participants’ Opinions Expressed During Cognitive Interviews of the Visual Analog Scale for the Interference of Menstrual Bleeding in Daily Activities

Second intermediary version	Participants’ opinions	Third intermediary version
INTERFERENCE OF MENSTRUAL BLEEDING IN DAILY ACTIVITIES—VISUAL ANALOGUE SCALE -	Generally, clear question. Interference, interfere, Impact, MB—good, appropriate, relevant vocabulary—emphasize severity of problem.	INTERFERENCE OF MENSTRUAL BLEEDING IN DAILY ACTIVITIES - VISUAL ANALOGUE SCALE –
Please assess the impact of your menstrual bleeding on your daily activities.	Patients understand “daily activities.” However, some preference for “daily life”/ everyday life instead of more formal “daily activities.”	Please assess the impact of your menstrual bleeding on your daily life/ activities.
Mark a vertical stroke on the following line to indicate how much your menstrual bleeding interferes in your daily activities.	Note: inverting question could sound more natural *i.e.*, “Indicate how much your menstrual bleeding interferes in your daily activities by drawing a line on the following line.”	Draw a vertical line on the following line to indicate how much your menstrual bleeding interferes in your daily life/activities.
Does not interfere in my daily activities at all	Minority reference to switching “in” to “with” in scale, as “more correct.” “Daily activities,” can also suggest very active sports, or “just pleasurable” activities to a minority. Adding “life” means it refers to any/all daily activities.	Does not interfere in my daily life/ activities at all.
Totally interferes in my daily activities	Minority reference to switching “in” to “with” in scale, as “more correct.” “Daily activities,” can also suggest very active sports, or “just pleasurable” activities to a minority. Adding “life” means it refers to any/all daily activities. Note: “Completely” can be used instead of “totally,” and can sound more natural, although they are almost synonymous.	Option 1: Totally interferes in my daily life/ activities. Option 2: Completely interferes in my daily life/ activities.

Overall, the SAMANTA-Q and the HMB-VAS tool were considered easy to read and quick to answer, with shorter questions being particularly favored for their clarity and ease of understanding. Participants valued the questionnaire’s concise length, while still finding it comprehensive, covering most of the issues related to HMB that they commonly encountered. The questions were well-received; participants considered them relevant and reflective of their experiences with HMB. It was widely agreed that the questionnaire offered a clear and accurate portrait of their condition to healthcare professionals.

Participants highlighted certain aspects that needed modification, which prompted adjustments to align the questionnaire language with the natural flow of English to improve question clarity and to minimize ambiguities. This was done because some phrases and expressions did not sound typically English or did not flow well in English. Minimal adjustments were implemented to facilitate reading and comprehension while preserving the original meaning and nuances from the Spanish version. Throughout this process, the fidelity and meaning of the original Spanish version were maintained. Overall, minor changes were applied (Supplementary Tables S1, S2, and S3). Following the analysis and interpretation of the cognitive interview results, the 3rd intermediary version was created (Tables 2–4).

**FIG. 2.**
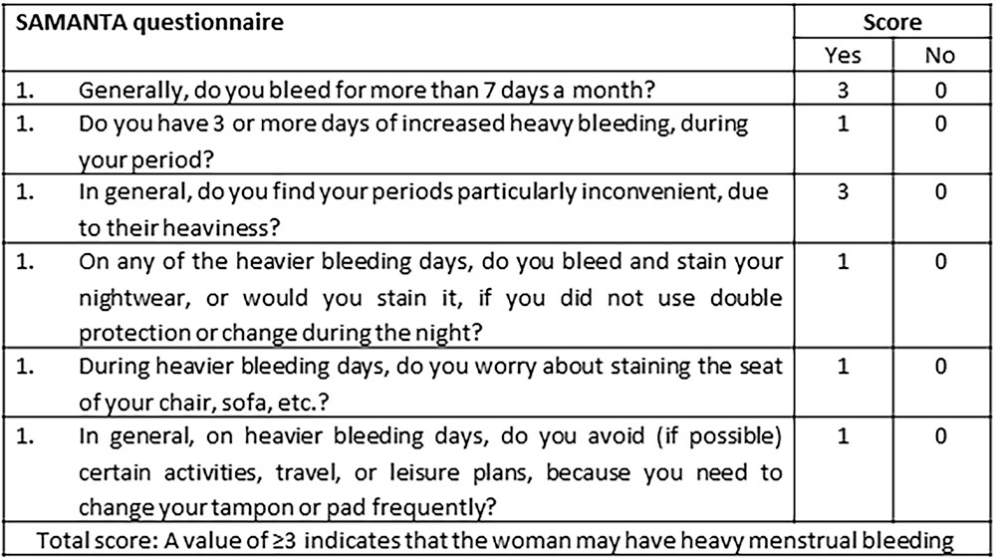
Final version of the linguistically validated British-English SAMANTA questionnaire.

**FIG. 3.**
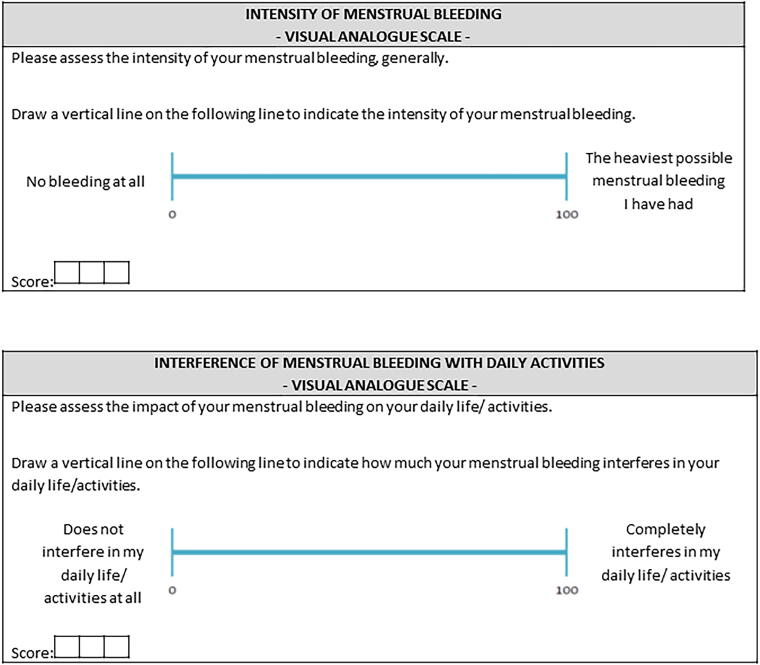
Final versions of the linguistically validated British-English HMB-VAS tool, including VAS-Int and VAS-Imp scales. VAS-Int, VAS for menstrual bleeding intensity; VAS-Imp, impact of heavy menstrual bleeding on activities of daily living.

To create the Final Version, a proofreading process was undertaken on the 3rd intermediary version to identify and rectify any typographical, grammatical, or other kind of errors or discrepancies compared to the original Spanish version. The final version of the SAMANTA-Q can be found in Figure 2, and the final version of the HMB-VAS tool, including VAS-Imp and VAS-Int, can be found in Figure 3.

## Discussion

The linguistic validation of the SAMANTA-Q and the HMB-VAS tool (VAS-Int and VAS-Imp) into British English represents a significant advancement in the field of women’s health, in the domain of HMB. This study meticulously adhered to the ISPOR guidelines for Translation and Cultural Adaptation of PROMs,^36^ to ensure that the translation process maintained the accuracy, reliability, and cultural relevance of the original Spanish instruments. The validation process followed the standardized procedure with rigorous methodology, resulting in the creation of user-friendly, hetero-administered tools to easily detect excessive menstrual blood loss and its impact on the QoL of British English-speaking women. As reported by Maneesriwongul,^39^ a comprehensive method involving translation and back-translation by bilingual or monolingual individuals ensures the highest quality in the translation process, as was done in our study. This methodology is supported by more than 4500 studies. During the forward translation process, no significant comprehension difficulties, or major differences between the chosen terms by both the back translators, were encountered in any of the questionnaires or scales.

The prevalence of HMB and its impact on womens’ QoL underscore the need for robust tools to assess this condition. HMB can have serious medical consequences and significantly affect daily life, impacting not only physical health but also psychological, social, and economic domains. However, barriers such as underreporting and normalization of HMB symptoms by affected women have historically hindered their seeking of appropriate care.^40,41^ Womens’ pain and discomfort during menstruation have often been dismissed, minimized, or overlooked, perpetuating stereotypes and taboos surrounding menstrual health. In addition, limited knowledge about what constitutes a “normal” menstrual cycle further complicates the situation.

The development and validation of the SAMANTA-Q and the HMB-VAS tool in English aimed to address these challenges by providing healthcare professionals with reliable instruments for evaluating HMB and its impact on patients’ lives. By offering patient-centred tools to measure menstrual blood loss, we can empower women to better articulate their experiences and seek the care they need. Addressing these attitudes and barriers is crucial to improve clinical outcomes and enhance QoL for individuals affected by HMB.

Besides the SAMANTA-Q, several survey instruments are available for HMB impact measurement in English, including unidimensional and multidimensional questionnaires. However, some are excessively long,^27–29^ culturally sensitive,^31^ or more focused on detecting associated hemostatic disorders than HMB’s impact on womens’ lives.^32^ The Menstrual Bleeding Questionnaire (MBQ) effectively distinguishes between women with and without HMB, addressing social embarrassment and behavioral changes related to menstrual bleeding. However, its 20-item length poses a burden on completion.^28^ In contrast, the SAMANTA-Q includes 6 questions, with concise format and binary response options (Yes/No), aligns with the need for accessible and time-efficient tools in the clinical setting. Its design facilitates a straightforward assessment of HMB, allowing for the rapid identification of women who may require further medical attention. SAMANTA’s real-time data collection minimizes recall bias and comprehensively captures the impact on daily activities, bridging gaps between physician assessments and patient experiences. This underscores the importance of appropriate instruments to quantify the symptomatic burden of HMB on QoL, considering both bleeding severity and its impact. The measurement of PROs is an area of HMB research because there are few multidimensional questionnaires (assessing symptoms and QoL assessment in combination).^28^ For this reason, the SAMANTA-Q and the HMB-VAS, which allow easy identification of women with HMB with a simple tool with high sensitivity and specificity, internal consistency, discriminatory capacity, and ease of use, are of special interest. The HMB-VAS tool complements this by offering a nuanced measure of the intensity and interference of menstrual bleeding on daily activities. The combination of these tools provides a comprehensive overview of the patient’s condition, which is essential to facilitate personalized care. The SAMIRA study, which measured the sensitivity to change of these tools, demonstrated their discriminatory benefits for the detection and monitoring of women with HMB.^37^ The performance of SAMANTA-Q and HMB-VAS derived from the SAMIRA study, where women highlighted a reduction in the SAMANTA-Q score, was associated with improvements in PROMs, including a shorter duration of menstrual bleeding. The impact of HMB encompass all aspects of health-related QoL, showing significant improvement across all domains of the SF-56 questionnaire with the reduction of SAMANTA-Q scores. Multidimensional questionnaires with outstanding reliability (such as SAMANTA-Q and HMB-VAS) are highly practical for clinicians, with excellent reliability and ease of use in daily clinical practice. The SAMANTA-Q and the HMB-VAS tool were designed to subjectively reflect womens’ experiences and should be used to complement clinical data. Besides the clear benefit of evaluating impact on QoL, the SAMANTA-Q and the HMB-VAS tool bring “added value” by helping to link daily experiences to clinical practice.

This linguistic validation study has underscored the importance of cultural sensitivity in the development of PROMs. The cognitive debriefing process revealed the need for minor linguistic adjustments to ensure that the translated instruments resonated with British English-speaking women, thereby enhancing their applicability and acceptance. The incorporation of feedback from a diverse group of participants has contributed to the refinement of the tools, ensuring that they are representative of the experiences of women with HMB in the United Kingdom. Given the increasing diversity of cultures and languages in some countries, it is critical that culturally adapted and accessible instruments are available for all individuals. The linguistic validation of these instruments reinforces the connection between the instrument and the clinical reality, highlighting its usefulness and potential contribution to improving womens’ QoL. In fact, the SAMANTA-Q was used recently in a sociodemographic multinational cross-sectional study on HMB in different countries.^42^ In that study, a linguistically validated version was not used, but the questionnaire was translated verbatim into the language of the respective country of participation. It is undeniably important that reliable tools for HMB assessment are made available in different languages, and this study emphasizes the urgent need for easy-to-use instruments that are also linguistically validated. This will ensure accurate and comparable results in the assessment of HMB.

Finally, the importance of validating these measurement tools into English rely not only on detecting HMB among British English-speaking women and its link with the clinical reality, but also on obtaining standardized health indexes in research. In medicine and health research, validating instruments in different languages is crucial as it enables the conduct of international multicenter comparative studies. Linguistic validation facilitates the globalization of clinical research, which is essential for scientific, cultural, economic, and humanistic advancement worldwide. International multicenter studies yield robust evidence, enhancing generalizability and accelerating results, thereby improving patient outcomes. Hence, our aim with this linguistic validation is to offer support to researchers and English-speaking communities. We believe it is essential to disseminate the SAMANTA-Q through the standard channels of gynecology.

The linguistic validation of PROMs such as SAMANTA-Q and the HMB-VAS tools in clinical research and healthcare can significantly impacts women’s health in the following ways:

a) Enables more accurate and comprehensive assessment of HMB and its impact on QoL, leading to precise diagnosis and individualized interventions.
b) Validated questionnaires improve doctor–patient communication, allowing patients to express their experiences clearly, improving the assessment of HMB severity and its impact on patient lives.
c) Empowers women to self-assess their condition, raising awareness and encouraging medical care-seeking behavior.
d) Permits the use of these instruments in international multicenter studies, ensuring the collection of comparable and reliable data across different linguistic and cultural contexts.
e) Data collected through these instruments can inform public health policies and programs aimed at improving detection, treatment, and education about HMB.
f) Clinical researchers can use these tools to evaluate the effectiveness of treatments and interventions, potentially leading to the development of new therapies and management strategies for HMB.
g) PROMs value the patient’s perspective in research and clinical practice, essential for patient-centered care to empower patients to actively take part in their medical care.
h) A validated version in British English is a more accessible starting point for validation into other languages.

In summary, the linguistic validation of these instruments is a step toward health equity, ensuring that women from different linguistic backgrounds have access to high quality assessment tools.

### Strengths and limitations

A key strength of this study was that forward translators were professional native British English-speaking linguists, and back translators were professional native Spanish-speaking linguists. Back translators were also blinded to the original Spanish questionnaire, ensuring the quality of the process. The interviewer was an expert on developing linguistic instruments, with deep knowledge of both languages, and extensive expertise in linguistic validation and interview and cognitive debriefing. Regarding cognitive debriefing, according to the COnsensus-based Standards for the selection of health Measurement INstruments (COSMIN) group, a sample size of ≥7 participants is very good for qualitative studies. In the present study a sample of 12 participants was included, thus being a reliable and adequate sample size to draw conclusions.^43^ All included participants were diagnosed with HMB and were familiar with the terminology associated with HMB. In addition, participants were balanced by educational levels, thus avoiding the potential influence of educational backgrounds on the results. By way of study limitations, the present study included only British English native speakers and interviewers; therefore, the resulting British English SAMANTA-Q and HMB-VAS tool will only be valid to be used with British English speakers.

The results of this linguistic validation study pertain solely to British English speakers and might not be universally applicable to other English-speaking populations or in diverse cultural contexts. Although SAMANTA-Q and HMB-VAS instruments are relevant and comprehensible to British English-speaking women, further validation studies may be required to tailor them for use by other linguistic and cultural groups. In addition, the psychometric properties of this English version have not been evaluated. While it is assumed that these properties are maintained from the original scale, given that the linguistic validation process followed established guidelines, it would be valuable to empirically assess them in an English-speaking population.

### Future research

Tools recognizing patients’ perspectives are crucial for a comprehensive evaluation of disease impact; yet, they are under-researched. There are limited available instruments to support clinical decision making, to assess treatments’ effectiveness, or to evaluate satisfaction among women with HMB. Potential benefits of future instruments rely on rigorous development and validation through well-designed international studies.

## Conclusion

This linguistic validation study marks a significant milestone in women’s health by providing a meticulously crafted British English version of the SAMANTA-Q, and of the HMB-VAS (VAS-Int and VAS-Imp) tool. Ensuring both conceptual and linguistic equivalence to the originals, these newly developed tools support clinical efforts to improve the QoL of English-speaking women with HMB. Furthermore, availability of such tools in multiples languages will not only facilitate international comparability but also will broaden the scope and impact of future research efforts on a global scale.

## Abbreviations Used

CEIm: Ethics Committee for research with medicinal products
COSMIN: COnsensus-based Standards for the selection of health Measurement INstruments
GCSE: General Certificate of Secondary Education
HB: Heavy Bleeding
HMB: Heavy Menstrual Bleeding
HMB-VAS: Heavy Menstrual Bleeding-Visual Analog Scale
ISPOR: International Society for Health Economics and Outcomes Research
MB: Menstrual Bleeding
MBQ: Menstrual Bleeding Questionnaire
PROs: Patient-Reported Outcomes
PROMs: Patient-Reported Outcome Measures
QoL: Quality of Life
SAMANTA-Q: SAMANTA Questionnaire
SD: Standard Desviation
SF-56: Short Form-56
VAS: Visual Analog Scale
VAS-Imp: Visual Analog Scale for its interference with daily activities
VAS-Int: Visual Analog Scale for menstrual bleeding intensity
