# Supplementary material for: Linguistic Validation of a British-English Version of the SAMANTA Questionnaire and HMB-VAS Tool: A Step Toward Improved Diagnosis of Heavy Menstrual Bleeding
Source: Womens Health Rep (New Rochelle). 2024 Dec 10;5(1):1017–31. doi: 10.1089/whr.2024.0061 (PMC11693961; doi:10.1089/whr.2024.0061)
Supplement: Supplementary Table S1 [file whr.2024.0061_supplementarytables1.pdf]

SUPPLEMENTARY TABLES

Supplementary Table S1. First, second and third intermediary versions of the SAMANTA questionnaire.

Original Spanish wording	Froward translation	First intermediary version	Second intermediary version	Third intermediary version
**Title**<br><br>Cuestionario SAMANTA	Both forward translations are identical.	SAMANTA questionnaire	SAMANTA questionnaire	SAMANTA questionnaire
**Item 1**<br><br>¿Sangra durante más de 7 días al mes?	**T1.** Do you bleed for more than 7 days a month?<br><br>**T2.** Do you bleed for more than 7 days every month?	Do you bleed for more than 7 days every month?	Do you bleed for more than 7 days every month?	Generally, do you bleed for more than 7 days every a month?
**Item 2**<br><br>¿Tiene 3 o más días de sangrado más abundante durante su menstruación?	Both forward translations are identical.	Do you have 3 or more days of heavier bleeding during your period?	Do you have 3 or more days of increased heavy bleeding during your period?	Do you have 3 or more days of increased heavy bleeding, during your period?
**Item 3**<br>En general, ¿su regla le resulta especialmente molesta debido a su abundancia?	**T1.** Generally speaking, is your period particularly irritating due to being heavy?<br><br>**T2.** In general, do you find your periods particularly inconvenient because of their intensity?	In general, do you find your periods particularly inconvenient because of their intensity?	In general, do you find your periods particularly inconvenient due to their heaviness?	In general, do you find your periods particularly inconvenient, due to their heaviness?
**Item 4**<br><br>¿En alguno de los días de sangrado más abundante mancha la ropa por las noches; o la mancharía si no usase doble protección o se cambiase durante la noche?	**T1.** On any of the days of heaviest bleeding, does it stain your clothes at night? Or would it stain your clothes if you did not use double sanitary protection or change your sanitary protection overnight?<br><br>**T2.** On any of the heavier bleeding days, do you leak through your clothes at night, or would you leak through them if you did not use double protection or change during the night?	On any of the heavier bleeding days, do you leak through your clothes at night, or would you leak through them if you did not use double protection or change during the night?	On any of the heavier bleeding days, do you bleed and stain your clothes during the nights, or would you stain them if you did not use double protection or change during the nights?	On any of the heavier bleeding days, do you bleed and stain your nightwear during the nights, or would you stain it, if you did not use double protection or change during the nights.

**Item 5**<br><br>¿Durante los días de sangrado más abundante le preocupa manchar el asiento de su silla, sofá, etc.?	**T1.** On the days of heaviest bleeding, do you worry about staining your chair, sofa, etc.?<br><br>**T2.** During heavier bleeding days, do you worry about staining your chair, sofa, etc.?	During heavier bleeding days, do you worry about leaking through onto your chair, sofa, etc.?	During heavier bleeding days, do you worry about staining the seat of your chair, sofa, etc.?	During heavier bleeding days, do you worry about staining the seat of your chair, sofa, etc.?
**Item 6**<br><br>En general, ¿en los días de sangrado más abundante, evita (en la medida de lo posible) algunas actividades, viajes o planes de ocio porque debe cambiarse frecuentemente el tampón o la compresa?	**T1.** In general, on the days of heaviest bleeding, do you avoid (as far as possible) certain activities, trips or leisure plans because you have to change your tampon or sanitary towel frequently?<br><br>**T2.** In general, on heavier bleeding days, do you avoid (as much as possible) some activities, travel or leisure plans because you have to change your tampon or pad frequently?	In general, on heavier bleeding days, do you avoid (as much as possible) some activities, travel or leisure plans because you have to change your tampon or pad frequently?	In general, on heavier bleeding days, do you avoid (as much as possible) some activities, travel or leisure plans because you need to change your tampon or pad frequently?	In general, on heavier bleeding days, do you avoid (as much as possible if possible) some certain activities, travel, or leisure plans, because you need to change your tampon or pad frequently?
**Explanation**<br>Puntuación total: Un valor ≥ 3 indica que la mujer puede tener sangrado menstrual abundante	**T1.** Total score: A score ≥ 3 suggests that the woman may have heavy menstrual bleeding<br><br>**T2.** Total score: A value of ≥3 indicates that the woman may have heavy menstrual bleeding	Total score: A value of ≥3 indicates that the woman may have heavy menstrual bleeding	Total score: A value of ≥3 indicates that the woman may have heavy menstrual bleeding	Total score: A value of ≥3 indicates that the woman may have heavy menstrual bleeding
**Score**<br><br>Puntuación (Sí/No)	Both forward translations are identical.	Score (Yes/no)	Score (Yes/no)	Score (Yes/no)
