# Supplementary material for: Linguistic Validation of a British-English Version of the SAMANTA Questionnaire and HMB-VAS Tool: A Step Toward Improved Diagnosis of Heavy Menstrual Bleeding
Source: Womens Health Rep (New Rochelle). 2024 Dec 10;5(1):1017–31. doi: 10.1089/whr.2024.0061 (PMC11693961; doi:10.1089/whr.2024.0061)
Supplement: Supplementary Table S2 [file whr.2024.0061_supplementarytables2.pdf]

Supplementary Table S2. First, second and third intermediary versions of the VAS for the intensity of menstrual bleeding (VAS-Int).

**Original Spanish wording**	**Translation**	**First intermediary version**	**Second intermediary version**	**Third intermediary version**
**Title**<br><br>INTENSIDAD DEL SANGRADO MENSTRUAL<br><br>- ESCALA VISUAL ANALÓGICA -	**T1.** MENSTRUAL BLEEDING INTENSITY – VISUAL ANALOGUE SCALE –<br><br>**T2.** INTENSITY OF MENSTRUAL BLEEDING – VISUAL ANALOGUE SCALE –	INTENSITY OF MENSTRUAL BLEEDING<br><br>- VISUAL ANALOGUE SCALE-	INTENSITY OF MENSTRUAL BLEEDING - VISUAL ANALOGUE SCALE -	INTENSITY OF MENSTRUAL BLEEDING<br>VISUAL ANALOGUE SCALE
**Item 1**<br><br>Por favor, valore la intensidad de su sangrado menstrual.	Both forward translations are identical.	Please rate the intensity of your menstrual bleeding.	Please assess the intensity of your menstrual bleeding.	Please assess the intensity of your menstrual bleeding, generally
**Item 2**<br><br>Marque una raya vertical sobre la línea siguiente indicando la intensidad de su sangrado menstrual.	**T1.** Draw a vertical line on the horizontal line below to indicate the intensity of your menstrual bleeding.<br><br>**T2.** Draw a vertical line on the line below to indicate the intensity of your menstrual bleeding.	Draw a vertical line on the line below to indicate the intensity of your menstrual bleeding.	Mark a vertical stroke on the following line to indicate the intensity of your menstrual bleeding.	Option 1:<br>Draw a vertical line on the following line to indicate the intensity of your menstrual bleeding.<br>Option 2:<br>Draw a vertical line on the following line to indicate the intensity of your menstrual bleeding.
**Item 3**<br><br>Ningún sangrado en absoluto	Both forward translations are identical.	Not bleeding at all	Not bleeding at all	0' Not bleeding at all
**Item 4**<br><br>El sangrado más abundante posible que he visto	**T1.** The heaviest bleeding I have ever had<br><br>**T2.** The heaviest possible bleeding I have seen	The heaviest possible bleeding I have seen	The heaviest possible bleeding I have seen	Option 1:<br>'100' The heaviest menstrual possible bleeding possible<br>Option 2:<br>'100' The heaviest possible menstrual bleeding I have had seen

**Item 5**<br><br>Puntuación (0-100)	Both forward translations are identical.	Score (0-100)	Score (0-100)	Score (0-100)

505
