# Supplementary material for: Linguistic Validation of a British-English Version of the SAMANTA Questionnaire and HMB-VAS Tool: A Step Toward Improved Diagnosis of Heavy Menstrual Bleeding
Source: Womens Health Rep (New Rochelle). 2024 Dec 10;5(1):1017–31. doi: 10.1089/whr.2024.0061 (PMC11693961; doi:10.1089/whr.2024.0061)
Supplement: Supplementary Table S3 [file whr.2024.0061_supplementarytables3.pdf]

Supplementary Table S3. First, second and third intermediary versions of the VAS for interference of menstrual bleeding in daily activities (VAS-Imp).

Original Spanish wording	Translation	First intermediary version	Second intermediary version	Third intermediary version
**Title**<br>INTERFERENCIA DEL SANGRADO MENSTRUAL EN LAS ACTIVIDADES COTIDIANAS<br><br>- ESCALA VISUAL ANALÓGICA -	**T1.** HOW YOUR MENSTRUAL BLEEDING AFFECTS YOUR DAILY ACTIVITIES – VISUAL ANALOGUE SCALE<br><br>**T2.** INTERFERENCE OF MENSTRUAL BLEEDING WITH DAILY ACTIVITIES – VISUAL ANALOGUE SCALE –	INTERFERENCE OF MENSTRUAL BLEEDING WITH DAILY ACTIVITIES<br><br>- VISUAL ANALOGUE SCALE	INTERFERENCE OF MENSTRUAL BLEEDING IN DAILY ACTIVITIES - VISUAL ANALOGUE SCALE -	INTERFERENCE OF MENSTRUAL BLEEDING IN DAILY ACTIVITIES<br>- VISUAL ANALOGUE SCALE<br>–
**Item 1**<br><br>Por favor, valore el impacto que tiene su sangrado menstrual en sus actividades cotidianas.	**T1.** Please rate to what extent your menstrual bleeding affects your daily activities.<br>**T2.** Please rate the impact of your menstrual bleeding on your daily activities.	Please rate the impact of your menstrual bleeding on your daily activities.	Please assess the impact of your menstrual bleeding on your daily activities.	Please assess the impact of your menstrual bleeding on your daily life/ activities.
**Item 2**<br><br>Marque una raya vertical sobre la línea siguiente indicando en qué medida interfiere su sangrado menstrual en sus actividades cotidianas.	**T1.** Draw a vertical line on the horizontal line below to indicate how much your menstrual bleeding interferes with your daily activities.<br><br>**T2.** Draw a vertical line on the line below to indicate how much your menstrual bleeding interferes with your daily activities.	Draw a vertical line on the line below to indicate how much your menstrual bleeding interferes with your daily activities.	Mark a vertical stroke on the following line to indicate how much your menstrual bleeding interferes in your daily activities.	Draw a vertical line on the following line to indicate how much your menstrual bleeding interferes in your daily life/activities.
**Item 3**	**T1.** No effect at all on my daily activities	Does not interfere with my daily activities at all	Does not interfere in my daily activities at all	Does not interfere in my daily life/ activities at all.

No interfiere en absoluto en mis actividades cotidianas	**T2.** Does not interfere with my daily activities at all			
**Item 4**<br><br>Interfiere totalmente con mis actividades cotidianas	**T1.** The greatest possible effect on my daily activities<br><br>**T2.** Completely interferes with my daily activities	Completely interferes with my daily activities	Totally interferes in my daily activities	Option 1:<br>Totally interferes in my daily life/ activities.<br>Option 2:<br>Completely interferes in my daily life/ activities.
**Item 5**<br>Puntuación (0-100)	Both forward translations are identical.	Score (0-100)	Score (0-100)	Score (0-100)

508
