# Supplementary material for: Linguistic Validation of a British-English Version of the SAMANTA Questionnaire and HMB-VAS Tool: A Step Toward Improved Diagnosis of Heavy Menstrual Bleeding
Source: Womens Health Rep (New Rochelle). 2024 Dec 10;5(1):1017–31. doi: 10.1089/whr.2024.0061 (PMC11693961; doi:10.1089/whr.2024.0061)
Supplement: Supplementary Appendix S1 [file whr.2024.0061_supplementaryappendixsa1.pdf]

Appendix 1

Original version of SAMANTA questionnaire and heavy menstrual bleeding visual analogue scale (HMB-VAS) tool.

<u>SAMANTA questionnaire in Spanish</u>

**SAMANTA questionnaire**	**Score**	
	Yes	No
1. ¿Sangra durante más de 7 días al mes?	3	0
2. ¿Tiene 3 o más días de sangrado más abundante durante su menstruación?	1	0
3. En general, ¿su regla le resulta especialmente molesta debido a su abundancia?	3	0
4. ¿En alguno de los días de sangrado más abundante mancha la ropa por las noches; o la mancharía si n usase doble protección o se cambiase durante la noche?	1	0
5. ¿Durante los días de sangrado más abundante le preocupa manchar el asiento de su silla, sofá, etc.?	1	0
6. En general, ¿en los días de sangrado más abundante, evita (en la medida de lo posible) algunas actividades, viajes o planes de ocio porque debe cambiarse frecuentemente el tampón o la compresa?	1	0
Puntuación total: Un valor ≥ 3 indica que la mujer puede tener sangrado menstrual abundante		

HMB-VAS tool in Spanish

INTENSIDAD DEL SANGRADO MENSTRUAL
- ESCALA VISUAL ANALÓGICA -

Por favor, valore la intensidad de su sangrado menstrual.

Marque una raya vertical sobre la línea siguiente indicando la intensidad de su sangrado menstrual.

Ningún sangrado en absoluto	0 ——————————————— 100	El sangrado más abundante posible que he visto

Puntuación: ☐☐☐

INTERFERENCIA DEL SANGRADO MENSTRUAL EN LAS ACTIVIDADES COTIDIANAS
- ESCALA VISUAL ANALÓGICA -

Por favor, valore el impacto que tiene su sangrado menstrual en sus actividades cotidianas.

Marque una raya vertical sobre la línea siguiente indicando en qué medida interfiere su sangrado menstrual en sus actividades cotidianas.

No interfiere en absoluto en mis actividades cotidianas	0 ——————————————— 100	Interfiere totalmente con mis actividades cotidianas

Puntuación: ☐☐☐
