# Supplementary material for: Linguistic Validation of a British-English Version of the SAMANTA Questionnaire and HMB-VAS Tool: A Step Toward Improved Diagnosis of Heavy Menstrual Bleeding
Source: Womens Health Rep (New Rochelle). 2024 Dec 10;5(1):1017–31. doi: 10.1089/whr.2024.0061 (PMC11693961; doi:10.1089/whr.2024.0061)
Supplement: Supplementary Appendix S2 [file whr.2024.0061_supplementaryappendixsa2.pdf]

Appendix 2

Cognitive interview guide

Instructions

The context of the project:

The global aim of this project is to validate a tool, a questionnaire to be fulfilled by women, to assess the intensity of your menstrual bleeding and its impact on daily activities.

The aim of the present interview is to assess whether the questionnaire asks about relevant questions, in a manner that is well-understood and clear to all respondents.

Material to be tested: questionnaire.

The aims of the interview are:

1. To evaluate the understanding and acceptability of the instructions, questions and answer choices contained in the test questionnaire.
2. To evaluate the understanding of the information contained in the information sheet.
3. To identify instructions, questions and/or response choices that are problematic or not relevant, and determine the reason(s) why.
4. To find solutions to improve the formulation of the instructions, questions and/or answer choices.
5. To determine if one or several item(s) is(are) missing, redundant or inappropriate.

The roles of the interviewer are:

1. **To schedule** an appointment for an interview with the participant.
2. **To perform** the interview following this interview guide.
3. **To reassure** the participant of confidentiality: The participant's name, contact information and questionnaire answers will not be provided to anyone not involved with this project. The participant's responses will not be connected to their name.
4. **To prompt the participant to speak and to express their feelings about the questionnaire,** plus in the cognitive debriefing form note what the participant subconsciously expresses (e.g. facial expressions indicating rejection or acceptance). For this purpose, a list of domains and examples of probes will be used (See Appendix 1). Probes examples are notes for the interviewer. As such, they should be used if the participant does not provide a sufficient amount of information or if the domain we want to assess (e.g. comprehension) is not well covered with their response. Please make sure that all points are covered.
5. **To encourage** the participant to express their feelings about each question, to express the questions in their own words and to give honest responses about the wording and appropriateness.

Introduction to cognitive interviewing methodology

Cognitive interviewing will be used to conduct the interviews; more questions will also be specifically asked for some items of the questionnaire.

Cognitive interviewing is a tool for improving questionnaire design and content. Cognitive interviewing will focus on the questionnaire rather than on the collection process of data, paying particular interest to the mental process used by the participant when answering a questionnaire.

Cognitive interviewing is a qualitative tool and is by nature flexible. It is composed of two main techniques:

- Thinking aloud
- Cognitive probes

In the interview, participants would be asked to complete a “think aloud” exercise. This involves the participant completing the questionnaire and being asked to speak aloud their thoughts as they read the question and any instructions and choose a response. Following the “think aloud” process, full cognitive debriefing will be conducted, in which the participants are asked detailed questions in case the domains had not been explored:

- about their understanding of the meaning of the questions and response options.
- about how well the instrument reflects their experience.
- about whether the response given by the participant is well thought through or just randomly answered.
- whether the question and response choices are easy to understand and relevant.
- if they would choose to reword the questions (in case of an incomprehension).
- if they think anything is missing that is important in their experience of the targeted symptoms.

Thinking aloud is not something natural for participants; prior to beginning the interview, it is therefore necessary to train participants in this method. For this purpose, the method proposed by Willis can be used (Willis GB, 1995). Briefly, this method consists in asking the participant to visualize the place where they live, and think about how many windows there are in that place. As they count the windows, the participant is asked to say aloud what they are seeing and thinking about it.

Once the participant is comfortable and has well understood the method, the cognitive interview can start.

The interview should be conducted as follows:

1. The interviews will be conducted by a researcher working at Adelphi or recruited by Adelphi.
2. <u>The interviews will be audio-recorded</u>. The records will serve as a source document.

   <u>Note to the interviewer</u>: The recording has to be checked at the end of each interview. In any case, extensive notes have to be taken during the interview.
   It is, however, essential that you summarize the overall interview (Appendix 2).

3. **Introduce** and explain the project to the participant. Thank her for agreeing to take part in the interview.
4. **Give the questionnaire to the participant and ask them to complete it.** Remind the participant that we are not interested so much in their responses as in the way the questions are asked. Let them know that we want their help to make the questionnaire better, so we do not mind, and in fact appreciate it if they point out problems with the questionnaire, or things that are unclear. Record the time it takes them to complete the questionnaire.
5. **Completion of the questionnaire:** Ask the participant to think aloud as they are responding to the questionnaire – what are they thinking about as they answer each question? If they seem to pause in confusion, probe on this 'you seemed to hesitate, why is that?'
6. **General impressions:** Ask the participant to give you their general feelings about the questionnaire (e.g., is it easy to understand, easy to answer, too long?).
7. **Titles and Instructions:** Go through the titles and instructions of the questionnaire to check:
   - Whether the instructions and titles were difficult to understand
   - Whether the instructions and titles were detailed enough
   - How the participant would word the instructions and title
8. **Questions and Answer choices:**
   - ➢ Go through each question of the questionnaire to check:
     - Whether the question was easy to understand
     - What the participant thinks the question is asking them
     - Whether the question is acceptable to the participant
     - How the participant would ask the question in their own words
   - ➢ Go through the answer choices for each question in the questionnaire to check:
     - Whether the answer choices were difficult to understand
     - Whether the answer choices were detailed enough
     - Whether the participant would reword the answer choices
     - Whether the answer choices go well with the question

1. Introduction

<u>*Notes to the interviewer:*</u>

- *In italics are the instructions and information for interviewers.*
- *Text within quotation marks is to be said by the interviewer. If not repeated word by word, the information should be covered.*
- *To conduct the cognitive interviewing a list of the domains that are to be covered and examples of probes are proposed at the end of the guide (Appendix 1).*
- *In red are the questions that are to be asked to the participants (e.g. "specific questions" and "alternatives").*
- *Items regarding "Questions and Response Choices" are particularly important and should be covered in-depth.*

THANK THE PARTICIPANT FOR AGREEING TO TAKE PART IN THIS STUDY

INTRODUCE YOURSELF

🗣 "I work for Adelphi is an international company that carries out studies on people's opinions focussed on health area. We also develop questionnaires for people to answer about their, health conditions, in general and how it affects them every day"

EXPLAIN THIS PARTICULAR PROJECT TO THE PARTICIPANT

🗣 "This questionnaire was developed to help women to assess the intensity of her menstrual bleeding and how it interferes in her daily activities"

EXPLAIN THE GOAL OF THE INTERVIEW AND THE INTERVIEW PROCESS TO THE PARTICIPANT

🗣 "I will ask you to fill in the questionnaire."

🗣 "I would like you to speak your thoughts out loud while you are completing the questionnaire, telling me what you are thinking about as you answer. If anything seems oddly worded to you or seems not to make sense, please let me know since we are trying to make the questionnaire the best it can possibly be and your opinion is important to us. After completing the questionnaire, I will ask you questions on each of the questions in the questionnaire. You may find these questions repetitive but we need to ask these questions to make sure we understand what you think about the questions and how the questions could be improved."

🗣 "The interview should last approximately 60 minutes and will be audio recorded, so please, try to think and speak aloud so that your comments can be heard".

🗣 "If the interview lasts for longer or if you do not want to continue the interview any longer, you may interrupt it at any time. You may also refuse to answer a question".

REASSURE THE PARTICIPANT OF CONFIDENTIALITY

🗣 "Be sure that anything you will say during this interview will be kept in strict confidence. Your name and information that could be used to identify you will not be revealed to any other person without your permission."

AUDIO RECORDING THE INTERVIEW

🗣 "The interview today will be recorded so that I can pay careful attention to what you say and to make certain I do not miss anything important in what you are telling me."

🗣 "The recording will not be shared with anyone other than the researchers involved in the study."

🗣 "Please be candid in your responses and don't be afraid to tell us what you think about the questionnaire – we would like your help to improve the wording of the questionnaire and will not be offended if you point out any problems with it."

BEFORE STARTING THE INTERVIEW

🗣 "Do you have any questions at this point?"

- *Remind the participant that she will have to fill in the questionnaire while speaking aloud, and that what you aim with this interview is her assessment of the way questions are formulated.*
- *Train the participant in the method proposed by Willis in order to make them more comfortable with the "thinking aloud" process. Once the participant is comfortable and have well understood the method, the cognitive interview can start.*
- *Obtain the participant's oral consent for the interview. This should also be present on the audio-tape if the participant agrees to have the interview tape-recorded.*

2. Questionnaire Completion

- Let participants know we are particularly interested in their understanding of the meaning of the questions.
- Instruct the participant to read aloud each question and to tell you what she is thinking as she reads aloud and as she answers the question. Make note of any hesitancy while reading or answering and probe on this at the time it occurs.
- Advise the participant to comment on or make notes in the margins as they complete the questionnaire if there is anything that is unclear, or if there is anything she feels should be added to or deleted from the questionnaire. This will help the participant remember any difficulties they may have encountered with a question when discussing it later in the interview.
- Emphasise to the participant that by helping the interviewer identify anything that is unclear in the questionnaire, the questionnaire can be improved.
- If the participant has any questions or comments regarding the questionnaire or completion of the questionnaire, discuss these fully.

Please note any comments you have about the participant's attitude, facial expressions, hesitancies, ease of reading/completion whilst they complete the questionnaire:

....

....

....

....

....

....

....

....

....

....

....

Time needed to complete the questionnaire: ________ minutes

3. General Impressions

- **What is your overall opinion of the questionnaire?** *(probe relevance, understanding, matching of response options with items – note that if these probes and the ones below were already covered in the read/think aloud portion, you do not need to cover these probes again)*

  Probe: What was difficult for you to answer?

  How was it difficult?

  How could it be made easier?

- **What is your opinion about the length of the questionnaire?**

- **Would you add any questions to the questionnaire? Which ones? Why?**

- **Would you delete any questions from the questionnaire? Which ones? Why? Are there any questions that do not seem important to you? Which ones? Why?**

- **What is your overall opinion of the general appearance (format / layout) of the questionnaire?**

What do you think about the order of the different sections of the questionnaire?

Do the questions follow on from each other well?

Probe: What do you think about the order of the questions within each section?

4. Introduction

- Did you read the introduction, skim the introduction, or skip right to the questions? Why?

- What does this introduction mean to you in your own words?

- Is there anything missing from the introduction?

- What would you change in the introduction, if anything?

5. Questions and Response Choices

Note to the interviewer: *The answer choices that are the same for several items of the questionnaire have only been included once in the guide, after the first relevant question. However, these answer choices should be tested as far as is reasonable with the respondent, for every item in the questionnaire, asking the following questions: 1. Are the answer choices relevant to the question? (i.e.: do the answer choices match the question?) - 2. Would you change any of the answer choices? Why? Why not?*

Section 1: INTENSITY OF MENTRUAL BLEEDING -VISUAL ANALOGUE SCALE –

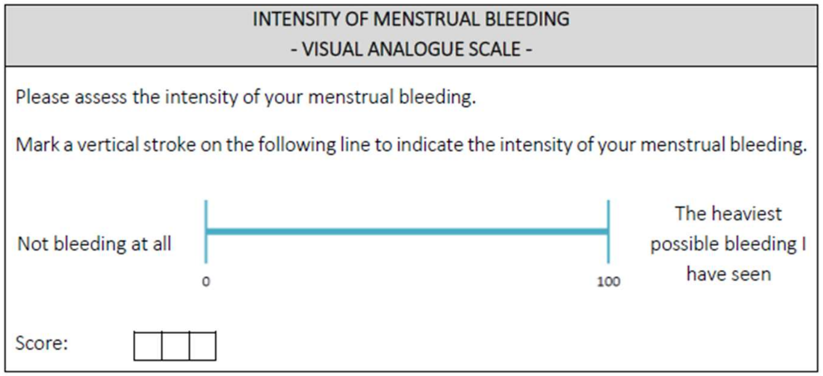
INTENSITY OF MENSTRUAL BLEEDING
- VISUAL ANALOGUE SCALE -

Please assess the intensity of your menstrual bleeding.

Mark a vertical stroke on the following line to indicate the intensity of your menstrual bleeding.

Score:

- What does this scale mean to you in your own words?
- What were you thinking when you answered?
- How did you complete the scale? Did you find it easy / difficult to complete this scale?
- Is there anything missing?
- What would you change, if anything?
- What does “assess” mean to you?
- What does “not bleeding at all” mean to you?
- What does “the heaviest possible bleeding I have seen” mean to you?

Section 2: INTERFERENCE OF MENSTRUAL BLEEDING IN DAILY ACTIVITIES -VISUAL ANALOGUE SCALE –

INTERFERENCE OF MENSTRUAL BLEEDING IN DAILY ACTIVITIES
- VISUAL ANALOGUE SCALE -

Please assess the impact of your menstrual bleeding on your daily activities.

Mark a vertical stroke on the following line to indicate how much your menstrual bleeding interferes in your daily activities.

Does not interfere in my daily activities at all	0 ——————————— 100	Totally interferes in my daily activities

Score: ☐☐☐

- What does this scale mean to you in your own words?
- What were you thinking when you answered?
- How did you complete the scale? Did you find it easy / difficult to complete this scale?
- Is there anything missing?
- What would you change, if anything?
- What does “assess” mean to you?
- What does “daily activities” mean to you?
- What does “not interfere at all” mean to you?
- What does “totally interferes” mean to you?

Section 3: SAMANTA questionnaire

Question 1	What does this question mean to you in your own words?	What were you thinking when you answered?
Do you bleed for more than 7 days every month?		
	What would you change in the question, if anything?	What does “bleed” mean to you?<br>What does “7 days every month” mean to you?
Response Choices	Do the answer choices match the question?	Would you change any of the response choices? Why? Why not?
YES / NO		
Question 2	What does this question mean to you in your own words?	What were you thinking when you answered?
Do you have 3 or more days of increased heavy bleeding during your period?	What would you change in the question, if anything?	What does “increased heavy bleeding” mean to you?<br>What does “heavy bleeding” mean to you?

Response Choices	Do the answer choices match the question?	Would you change any of the response choices? Why? Why not?
YES / NO		
Question 3	What does this question mean to you in your own words?	What were you thinking when you answered?
In general, do you find your periods particularly inconvenient due to their heaviness?		
	What would you change in the question, if anything?	What does “inconvenient” mean to you?<br><br>What does “period” mean to you?<br><br>What does “due to their heaviness” means to you?
Response Choices	Do the answer choices match the question?	Would you change any of the response choices? Why? Why not?
YES / NO		
Question 4	What does this question mean to you in your own words?	What were you thinking when you answered?
On any of the heavier bleeding days, do you bleed and stain your clothes during the nights, or would you stain them if you did not use double protection or change during the nights?		

	What would you change in the question, if anything?	What does "stain" mean to you?<br><br>What does "during the nights" mean to you?<br><br>What does "double protection" mean to you?
Response Choices	Do the answer choices match the question?	Would you change any of the response choices? Why? Why not?
YES / NO		
Question 5	What does this question mean to you in your own words?	What were you thinking when you answered?
During heavier bleeding days, do you worry about staining the seat of your chair, sofa, etc.?		
	What would you change in the question, if anything?	What does "heavier bleeding days" mean to you?<br><br>What does "worry" mean to you?
Response Choices	Do the answer choices match the question?	Would you change any of the response choices? Why? Why not?
YES / NO		
Question 6	What does this question mean to you in your own words?	What were you thinking when you answered?

In general, on heavier bleeding days, do you avoid (as much as possible) some activities, travel or leisure plans because you need to change your tampon or pad frequently?		
	What would you change in the question, if anything?	What does “avoid” mean to you?
Response Choices	Do the answer choices match the question?	Would you change any of the response choices? Why? Why not?
YES / NO		

Appendix 1: Domains, example of probes and objectives to be explored for the interview

<u>Note for the interviewer</u>:

- *The cognitive probes in the "questions" part of the Table below have to be asked systematically for each of the questions / Sentences of the questionnaire*
- *Comprehension is to be understood at two levels:*
    - *The first level corresponds to the overall comprehension of the question / instruction.*
    - *The second level corresponds to the case the question is followed with a list of options; the comprehension of each of these propositions by the participant will be assessed using the probes systematically.*
      In the case the participant has to choose between a "Yes / No", "Better / No difference / Worse" when answering a question, the cognitive probe in the "Response choice part of the Table below have to be asked to the participant.
- *If the participant expresses any reactions (oral, vocal) during the interview, please explore further the reason of this reaction*

RESTRICTED

Appendix 2: Notes of the interviewer to take once the interview is completed

Please, report your general impressions on the acceptability, understanding ease of use and emotions/reactions, difficulties encountered by the participant during the interview.

....

....

....

....

....

....

....

....

....

....

....

....

....

....

....

....

....

....

....

RESTRICTED
